# Systemic inflammation and modifiable risk factors for cognitive impairment in older persons: Findings from a British birth cohort

**DOI:** 10.1002/agm2.12044

**Published:** 2018-11-13

**Authors:** Alex Tsui, Marcus Richards, Daniel Davis

**Affiliations:** ^1^ MRC Unit for Lifelong Health and Ageing at UCL London UK

## Abstract

Serum pro‐inflammatory markers may contribute to dementia pathophysiology and cognitive impairment. In a population‐representative birth cohort, serum C‐reactive protein (CRP), interleukin‐6 (IL‐6), and white cell count (WCC) were measured at age 60‐64 years and cognition was assessed using the Addenbrooke's Cognitive Examination (ACE‐III) at age 69 years. Higher baseline CRP and IL‐6 were associated with lower ACE‐III scores, but associations were attenuated on adjustment for educational attainment, sex, and other modifiable life course factors. No associations were found for CRP, IL‐6, and WCC with visual search speed or verbal memory. In conclusion, the relationship between increased baseline systemic inflammation and poorer cognition in later life may be explained by, or share pathways with, education and other modifiable life course factors.

## 1 INTRODUCTION

Although neuroinflammation is a feature of Alzheimer's disease,^1^ the degree to which pathophysiology may be driven by systemic inflammation is unclear. For example, systemic illnesses with a pro‐inflammatory response may contribute to neurodegeneration and worsening dementia severity.^2^, ^3^, ^4^, ^5^ However, findings from epidemiological studies have been inconsistent^6^, ^7^, ^8^, ^9^, ^10^, ^11^; discrepancies may in part reflect complexities in modelling the temporal patterns of modifiable life course factors on both systemic inflammation and cognition. These factors include educational attainment,^12^ body mass index (BMI),^13^ alcohol intake,^14^ smoking, and exercise.^15^ Many of these exposures are time‐varying and so apparent associations with inflammation and cognition may be subject to reverse causation.

In this study, we set out to quantify the relationships among measures of systemic inflammation, modifiable life course factors known to be associated with cognitive impairment, and subsequent cognitive outcomes. We investigated this in a birth cohort with the advantage of accurate ascertainments of exposures across the whole life course, along with detailed assessment of cognition, to answer the following questions:

1. Is systemic inflammation prospectively associated with later cognitive impairment?
2. Is any association explained by modifiable lifestyle risk factors, such as early life educational attainment, body mass index, mid‐life exercise, alcohol consumption, or smoking?

## 2 METHODS

### 2.1 Population sample

The MRC National Survey for Health and Development (NSHD) is the oldest British birth cohort, following a sample of 5362 male and female participants born in 1 week in March 1946.^16^ During the 24th data collection in 2014‐2015, 2942 participants in the target sample living in England, Scotland, and Wales were contacted: 2453 (83.4%) returned a postal questionnaire and 2149 (73%) participants had a home visit by a research nurse.^17^ The target sample did not include participants no longer in the study (*n* = 2420): 957 (17.8% of the original sample) had already died, 620 (11.6%) had previously withdrawn from the study, 448 (8.3%) had emigrated and had no contact with the study, and 395 (7.4%) had been untraceable for more than 5 years.

### 2.2 Outcomes: General cognition

At age 69 years, the cognitive assessments comprised the Addenbrooke's Cognitive Examination, third version (ACE‐III), and verbal memory and visual search speed tasks, carried out by a research nurse according to the same standardized protocol as at earlier ages.^18^ A customized version of the ACE‐III was administered by iPad (Apple) using ACE Mobile (http://www.acemobile.org/). Of the 2149 participants visited by the research nurse, complete ACE‐III data were available for 1751 participants (81.5% of those who received a home visit).^17^ Verbal memory was assessed using a 15‐item word‐learning task, where each word was presented for 2 seconds. The score represents the total number of words correctly recalled over three identical trials (maximum 45). Visual search speed was assessed by crossing out the letters P and W, randomly embedded within a grid of other letters, as quickly and accurately as possible in 1 minute. The score represents the total number of letters searched (maximum 600).

### 2.3 Exposures: Serum inflammatory markers and covariates

At age 60‐64 years, participants had attended a clinic or had been visited by a research nurse, during which a blood sample had been taken. Aliquots were frozen and transferred to the MRC Human Nutrition Research Laboratory in Cambridge, UK, where analyses of inflammatory marker C‐reactive protein (CRP) and white blood cells were processed according to standardized protocols.^19^ Analyses of interleukin‐6 (IL‐6) were further undertaken by the British Heart Foundation Research Centre in Glasgow, UK.

During the home or clinic visits, waist circumference, height, and weight were measured using standardized protocols, and BMI was calculated (kg/m^2^). Participants were asked how many times in the last 4 weeks they had taken part in sports or vigorous activities, categorized as inactive (no episodes), less active (1‐4 exercise episodes/mo), and more active (≥5 exercise episodes/mo). Smoking history was prospectively self‐reported and calculated as pack‐years. Alcohol consumption was defined by frequency: four or more times per week; two to four times per week; two to four times per month; less than monthly; or a lifelong non‐drinker of alcohol. Educational qualifications by age 26 were categorized into none or less than ordinary secondary level (“O” levels usually taken at age 16); “O” levels; and advanced secondary level (“A” level) and higher. All data collected at age 60‐64 years were used to update corresponding information at earlier ages.

### 2.4 Statistical analysis

The complete sample with cognitive assessments at 69 years, serum inflammatory markers at 60‐64 years, and complete information on covariates data included 1008 participants. CRP, IL‐6, and white cell count (WCC) were divided into tertiles, consistent with the previous literature.^6^ All cognitive measures and continuous covariates were assessed for Gaussian distributions. Using a complete sample, associations between inflammatory exposures and cognition were estimated using univariate linear regression, then in turn adjusted for each selected covariate. All covariates that resulted in a change in model estimates when adjusted for were included in a final regression model (Table S1).

Linear regression was performed for all inflammation exposures and covariates with ACE‐III, then adjusted for educational attainment at age 26 years, then additionally adjusted for modifiable life course covariates. Similar models were estimated for visual search speed and verbal memory at 69 years, first adjusting for prior performance at 53 years, then additionally for educational attainment; and finally, additionally for modifiable life course covariates. STATA version 15.1 (StataCorp) was used for all analyses.

## 3 RESULTS

The prevalence of each exposure, including mean and standard deviations of inflammatory markers at age 60‐64 years, in relation to the sample followed at age 69 years is shown in Table 1. No significant sex differences were observed between each inflammation marker and individual covariates.

**Table 1:** Characteristics for NSHD participants completing ACE‐III at 69 y

Maximum sample with ACE‐III	NSHD age 60‐64 y		
	Men	Women	*P* value
*n*	841	910	
ACE‐III at 69 y	91.4 (5.64)	91.7 (6.13)	0.28
Mean visual search speed (SD)			
At 69 y	256 (74)	268 (74)	<0.01
At 53 y	273 (75)	289 (76)	<0.01
Mean verbal memory score (SD)			
At 69 y	21.1 (6.0)	23.2 (6.0)	<0.01
At 53 y	23.0 (6.2)	24.8 (6.3)	<0.01
Education			
<O levels	34.2	37.1	
O level	14.5	25.9	
≥A levels	51.3	36.9	<0.01
Body mass index (mean, SD)	27.9 (4.1)	27.9 (5.5)	0.94
Exercise activity at 60‐64 y (%)			
Never	61.3	59.3	
1‐4 times/wk	14.5	15.6	
≥5 times/wk	24.2	25.2	0.71
Smoking (pack‐years) at 60‐64 y			
Lifelong non‐smoker (%)	29.5	41.5	
1‐5 pack‐years (%)	17.3	20.9	
6‐20 pack‐years (%)	28.2	19.8	
>20 pack‐years (%)	25.1	17.8	<0.01
Alcohol intake at 68 y			
Never	17.0	29.0	
Less than monthly	5.2	8.0	
2‐4 times per month	13.1	17.9	
2‐4 times per week	28.1	22.9	
4 or more times per week	36.6	22.3	<0.01
CRP (median, IQR)	*n* = 685	*n* = 746	
	1.94 (0.78‐3.34)	2.14 (1.3‐3.71)	0.55
IL‐6 (median, IQR)	*n* = 680	*n* = 745	
	1.83 (1.26‐3.06)	1.82 (1.27‐2.89)	0.78
WCC (median, IQR)	*n* = 683	*n* = 749	
	5.9 (5.1‐7)	5.6 (4.8‐6.7)	<0.01

ACE‐III, Addenbrooke's Cognitive Examination, third version; CRP, C‐reactive protein; IL‐6, interleukin‐6; IQR, interquartile range; NSHD, National Survey for Health and Development; SD, standard deviation; WCC, white cell count.

### 3.1 Inflammation markers

There was an association between CRP and IL‐6 with ACE‐III scores in univariate analyses (maximum coefficients compared to reference: CRP −1.29 points, IL‐6 −1.27 points; *P* < 0.01). When adjusted for educational attainment, both associations were attenuated. Associations for all three inflammatory markers were further attenuated when fully adjusted for other modifiable life course factors (Table 2). No associations for CRP, IL‐6, and WCC with visual search speed or verbal memory were evident either in univariate or fully adjusted models (Tables S2 and S3).

**Table 2:** Linear regression analysis between ACE‐III at 69 y and serum inflammatory markers at 60‐64 y

ACE‐III	Univariate (complete)				Adjusted for educational attainment				Fully adjusted			
*n* = 1008	Coefficient	95% CI		*P* value	Coefficient	95% CI		*P* value	Coefficient	95% CI		*P* value
CRP at 60‐64 y												
1st tertile	Ref.				Ref.				Ref.			
2nd tertile	−0.72	−1.55	0.11		−0.43	−1.19	0.33		−0.29	−1.06	0.49	
3rd tertile	−1.26	−2.10	−0.41	0.01	−0.46	−1.24	0.32	0.42	−0.10	−0.97	0.76	0.76
IL‐6 at 60‐64 y												
1st tertile	Ref.				Ref.				Ref.			
2nd tertile	−0.96	−1.79	−0.14		−0.84	−1.59	−0.08		−0.56	−1.34	0.22	
3rd tertile	−1.27	−2.11	−0.42	<0.01	−0.73	−1.51	0.04	0.06	−0.32	−1.23	0.58	0.37
WCC at 60‐64 y												
1st tertile	Ref.				Ref.				Ref.			
2nd tertile	−0.50	−1.35	0.34	0.24	−0.24	−1.01	0.53		0.12	−0.66	0.90	
3rd tertile	−0.76	−1.60	0.07	0.19	−0.28	−1.04	0.48	0.73	0.32	−0.52	1.15	0.75
Educational attainment by 26 y												
<O levels	Ref.								Ref.			
O levels	3.98	3.11	4.85						3.60	2.72	4.49	
≥A levels	5.20	4.49	5.91	<0.01					4.70	3.94	5.47	<0.01
Sex	0.42	−0.27	1.11	0.23	0.62	−0.01	1.26	0.05	0.72	0.07	1.37	0.03
BMI at 60‐64 years (per SD)	−0.70	−1.07	−0.32	<0.01	−0.33	−0.68	0.02	0.06	−0.18	−0.55	0.20	0.35
Exercise at 60‐64 y												
Never	Ref.				Ref.				Ref.			
1‐4 times	1.44	0.44	2.44		0.53	−0.39	1.46		0.38	−0.55	1.31	
>5 times	2.08	1.27	2.89	<0.01	1.14	0.39	1.90	0.01	1.00	0.22	1.77	0.04
Smoking (pack‐years) at 60‐64 y												
Lifelong non‐smoker	Ref.				Ref.				Ref.			
1‐5 pack‐years	0.71	−0.23	1.65		0.83	−0.03	1.69		0.64	−0.23	1.51	
6‐20 pack‐years	−0.28	−1.18	0.63		0.28	−0.55	1.12		0.23	−0.62	1.09	
>20 pack‐years	−1.80	−2.76	−0.85	<0.01	−0.41	−1.31	0.48	0.09	−0.35	−1.30	0.60	0.26
Alcohol at 68 y												
Never	Ref.				Ref.				Ref.			
Less than monthly	−0.31	−1.86	1.25		−0.87	−2.31	0.56		−0.95	−2.38	0.48	
2‐4 times per month	1.40	0.28	2.53		0.55	−0.48	1.59		0.49	−0.55	1.53	
2‐4 times per week	1.41	0.42	2.40		0.53	−0.39	1.44		0.42	−0.52	1.35	
4 or more times per week	2.46	1.52	3.40	<0.01	1.12	0.23	2.00	0.03	1.09	0.18	2.01	0.03

ACE‐III, Addenbrooke's Cognitive Examination, third version; BMI, body mass index; CI, confidence interval; CRP, C‐reactive protein; IL‐6, interleukin‐6; WCC, white cell count.

### 3.2 Education attainment and lifestyle risk factors

Increased educational attainment, increased mid‐life exercise frequency, and lower mid‐life alcohol intake were prospectively associated with ACE‐III in fully adjusted models (fully adjusted: education 4.70 points [“A” level or higher vs less than “O” level, *P* < 0.01]; alcohol 1.1 points [four times per week vs none, *P* = 0.03]; and exercise 1.0 points [more than five times per week vs never, *P* = 0.04]). Increased BMI at 60‐64 years was associated with ACE‐III in univariate models (*P* < 0.01) but this association attenuated in the fully adjusted model (*P* = 0.35; Table 2). No associations were demonstrated between education or modifiable life course factors with later‐life visual search speed performance in fully adjusted models (Table S2). Educational attainment, exercise, alcohol intake, and smoking were associated with later life verbal memory performance in fully adjusted models (Table S3).

## 4 DISCUSSION

Our findings demonstrated that associations between mid‐life baseline IL‐6 and CRP with later life cognitive impairment were explained by educational attainment and modifiable life course factors. Mid‐life exercise, alcohol intake, and educational attainment were prospectively associated with domain‐specific impairment in verbal memory but not visual search speed, independently of mid‐life inflammation. Taken together, the relationship between systemic inflammation and later life cognition may be mediated by, or share pathways with, modifiable life course factors.

The literature on systemic inflammation, modifiable life course factors, and cognitive impairment has been inconsistent: While raised serum proinflammatory markers were prospectively associated with poorer cognitive performance in the Rotterdam,^9^ Framingham,^8^ and American Health, Aging, Body Composition cohorts,^10^ no associations were found in Dutch,^7^ Swedish,^11^ or other US cohorts.^7^, ^20^, ^21^, ^22^ These discrepancies likely reflect differences in study protocols, cohort ages, secular period, ethnicities, and degree of adjustment by confounders.

Our findings are unique in the following respects: First, participants were in their seventh decade, younger than other cohorts and perhaps at a critical period for when dementia pathophysiology is being initiated.^23^ In addition, the ACE‐III is a more detailed cognitive measure compared with briefer tests used in other studies, such as the Mini‐Mental State Examination. Nonetheless, our findings are limited by having a small range of serum pro‐inflammatory markers. In common with other cohort studies, our results may be subject to residual confounding and are specific to the population and era under study. Due to sample attrition, some data were missing and not at random. However, although we were unable to account for this in our model estimates, previous analyses within the NSHD cohort have shown only small differences between included participants and those excluded due to missing data.^19^ Multiple imputation of covariates, which can be intrinsically problematic, would likely result in minimal changes to the model.^24^ Unavailable confounders may have additionally contributed to cognitive changes: these included unrecorded comorbidities, such as atrial fibrillation, as well as changes in lifestyle behaviors between the studied ages of 60‐64 and 69 years, during transition from employment to retirement. Lastly, we assumed that levels of systemic inflammation would correspond to central inflammation across the whole population. This is unlikely to be the case because factors like blood‐brain barrier permeability are likely to vary between individuals,^25^ and even subclinical neurodegeneration may lead to microglial priming and consequent exaggeration of central inflammation.^26^

At least in NSHD, it appears the association between systemic inflammation and worse cognitive outcomes may be explained by: (a) pro‐inflammatory consequences of poor health behaviors (low physical activity, alcohol use, smoking) and/or (b) shared pathways between poor health behaviors and cognitive impairment. Our findings are consistent with a randomized trial that demonstrated that multi‐domain interventions—where exercise was a key component—resulted in cognitive benefits in older people.^27^ Furthermore, exercise has been associated with larger hippocampal volumes, increased gadolinium perfusion, and improved complex object recognition.^28^ Exercise is likely to be beneficial in reducing the vascular contributions to cognitive impairment from metabolic abnormalities, such as insulin resistance.

Despite these findings in the general population, there remains a case for investigating the relationship between systemic inflammation and neurodegeneration. Firstly, a wider panel of serum markers across the inflammatory cascade requires more detailed examination. Blood‐brain barrier dynamics linking serum and CNS inflammation also merits study, perhaps in the context of acute illnesses, such as delirium. Such a paradigm would investigate if different inflammatory burdens during acute illnesses contribute differently towards mechanisms in delirium and dementia and hence, variable dementia risks.^29^ An understanding of how systemic inflammation contributes to neurodegeneration, and their relation to already identified life course risk factors, might therefore implicate novel pathways towards modification of the course of Alzheimer's disease and other dementias.

## CONFLICT OF INTEREST

The authors have no conflict of interest to report.

## References

[1] Heneka MT , Carson MJ , El Khoury J , et al. Neuroinflammation in Alzheimer's disease. Lancet Neurol. 2015;14:388‐405. 25792098 10.1016/S1474-4422(15)70016-5 PMC5909703

[2] Davis DHJ , Skelly DT , Murray C , et al. Worsening cognitive impairment and neurodegenerative pathology progressively increase risk for delirium. Am J Geriatr Psychiatry. 2015;23:403‐415. 25239680 10.1016/j.jagp.2014.08.005 PMC4278840

[3] Skelly D , Griffin EW , Murray C , et al. Acute transient cognitive dysfunction and acute brain injury induced by systemic inflammation occur by dissociable IL‐1‐dependent mechanisms. Mol Psychiatry. 2018 [Epub ahead of print, 2018]. 10.1038/s41380-018-0075-8 PMC6510649 29875474

[4] Davis DH , Muniz‐Terrera G , Keage HA , et al. Association of delirium with cognitive decline in late life: A neuropathologic study of 3 population‐based cohort studies. JAMA Psychiatry. 2017;74:244. 28114436 10.1001/jamapsychiatry.2016.3423 PMC6037291

[5] Tsui A , Kuh D , Richards M , Davis D . Delirium symptoms are associated with decline in cognitive function between ages 53 and 69 years: Findings from a British Birth Cohort Study. Alzheimer's Dement. 2017;14:617‐622. 29161540 10.1016/j.jalz.2017.08.018 PMC5948100

[6] Singh‐Manoux A , Dugravot A , Brunner E , et al. Interleukin‐6 and C‐reactive protein as predictors of cognitive decline in late midlife. Neurology. 2014;83:486‐493. 24991031 10.1212/WNL.0000000000000665 PMC4141998

[7] Dik MG , Jonker C , Hack CE , Smit JH , Comijs HC , Eikelenboom P . Serum inflammatory proteins and cognitive decline in older persons. Neurology. 2005;64:1371‐1377. 15851726 10.1212/01.WNL.0000158281.08946.68

[8] Tan ZS , Beiser AS , Vasan RS , et al. Inflammatory markers and the risk of Alzheimer disease: The Framingham Study. Neurology. 2007;68:1902‐1908. 17536046 10.1212/01.wnl.0000263217.36439.da

[9] Engelhart MJ , Geerlings MI , Meijer J , et al. Inflammatory proteins in plasma and the risk of dementia: The Rotterdam Study. Arch Neurol. 2004;61:668‐672. 15148142 10.1001/archneur.61.5.668

[10] Yaffe K , Lindquist K , Penninx BW , et al. Inflammatory markers and cognition in well‐functioning African‐American and white elders. Neurology. 2003;61:76‐80. 12847160 10.1212/01.wnl.0000073620.42047.d7

[11] Sundelof J , Kilander L , Helmersson J , et al. Systemic inflammation and the risk of Alzheimer's disease and dementia: A prospective population‐based study. J Alzheimer's Dis. 2009;18:79‐87. 19542629 10.3233/JAD-2009-1126

[12] Richards M , Sacker A . Lifetime antecedents of cognitive reserve. J Clin Exp Neuropsychol. 2003;25:614‐624. 12815499 10.1076/jcen.25.5.614.14581

[13] Albanese E , Hardy R , Wills A , Kuh D , Guralnik J , Richards M . No association between gain in body mass index across the life course and midlife cognitive function and cognitive reserve–the 1946 British Birth Cohort study. Alzheimer's Dement. 2012;8:470‐482. 22858531 10.1016/j.jalz.2011.09.228 PMC3778923

[14] Richards M , Hardy R , Wadsworth ME . Alcohol consumption and midlife cognitive change in the British 1946 Birth Cohort Study. Alcohol Alcohol. 2005;40:112‐117. 15582985 10.1093/alcalc/agh126

[15] Cadar D , Pikhart H , Mishra G , Stephen A , Kuh D , Richards M . The role of lifestyle behaviors on 20‐year cognitive decline. J Aging Res. 2012;2012:304014. 22988508 10.1155/2012/304014 PMC3440944

[16] Kuh D , Pierce M , Adams J , et al. Cohort profile: Updating the cohort profile for the MRC National Survey of Health and Development: A new clinic‐based data collection for ageing research. Int J Epidemiol. 2011;40:e1‐e9. 21345808 10.1093/ije/dyq231 PMC3043283

[17] Kuh D , Wong A , Shah I , et al. The MRC National Survey of Health and Development reaches age 70: Maintaining participation at older ages in a birth cohort study. Eur J Epidemiol. 2016;31:1135‐1147. 27995394 10.1007/s10654-016-0217-8 PMC5206260

[18] Richards M , Shipley B , Fuhrer R , Wadsworth ME . Cognitive ability in childhood and cognitive decline in mid‐life: Longitudinal Birth Cohort Study. BMJ. 2004;328:552. 14761906 10.1136/bmj.37972.513819.EE PMC381045

[19] Elhakeem A , Murray ET , Cooper R , Kuh D , Whincup P , Hardy R . Leisure‐time physical activity across adulthood and biomarkers of cardiovascular disease at age 60–64: A prospective cohort study. Atherosclerosis. 2018;269:279‐287. 29180005 10.1016/j.atherosclerosis.2017.11.019 PMC5825380

[20] Wilson CJ , Cohen HJ , Pieper CF . Cross‐linked fibrin degradation products (D‐dimer), plasma cytokines, and cognitive decline in community‐dwelling elderly persons. J Am Geriatr Soc. 2003;51:1374‐1381. 14511156 10.1046/j.1532-5415.2003.51454.x

[21] Hsu PF , Pan WH , Yip BS , Chen RC , Cheng HM , Chuang SY . C‐reactive protein predicts incidence of dementia in an elderly Asian community cohort. J Am Med Dir Assoc. 2017;18:277.e7‐277.e11. 10.1016/j.jamda.2016.12.006 28159467

[22] Miwa K , Okazaki S , Sakaguchi M , Mochizuki H , Kitagawa K . Interleukin‐6, interleukin‐6 receptor gene variant, small‐vessel disease and incident dementia. Eur J Neurol. 2016;23:656‐663. 26725994 10.1111/ene.12921

[23] Rawle MJ , Davis D , Bendayan R , Wong A , Kuh D , Richards M . Apolipoprotein‐E (Apoe) epsilon4 and cognitive decline over the adult life course. Transl Psychiatry. 2018;8:18. 29317609 10.1038/s41398-017-0064-8 PMC5802532

[24] Davis DH , Kreisel SH , Muniz Terrera G , et al. The epidemiology of delirium: Challenges and opportunities for population studies. Am J Geriatr Psychiatry. 2013;21:1173‐1189. 23907068 10.1016/j.jagp.2013.04.007 PMC3837358

[25] Takeda S , Sato N , Morishita R . Systemic inflammation, blood‐brain barrier vulnerability and cognitive/non‐cognitive symptoms in Alzheimer disease: Relevance to pathogenesis and therapy. Front Aging Neurosci. 2014;6:171. 25120476 10.3389/fnagi.2014.00171 PMC4114193

[26] Cunningham C , MacLullich AM . At the extreme end of the psychoneuroimmunological spectrum: Delirium as a maladaptive sickness behaviour response. Brain Behav Immun. 2013;28:1‐13. 22884900 10.1016/j.bbi.2012.07.012 PMC4157329

[27] Ngandu T , Lehtisalo J , Solomon A , et al. A 2 year multidomain intervention of diet, exercise, cognitive training, and vascular risk monitoring versus control to prevent cognitive decline in at‐risk elderly people (FINGER): A randomised controlled trial. Lancet. 2015;385:2255‐2263. 25771249 10.1016/S0140-6736(15)60461-5

[28] Maass A , Duzel S , Goerke M , et al. Vascular hippocampal plasticity after aerobic exercise in older adults. Mol Psychiatry. 2015;20:585‐593. 25311366 10.1038/mp.2014.114

[29] Davis DH , Muniz Terrera G , Keage H , et al. Delirium is a strong risk factor for dementia in the oldest‐old: A population‐based cohort study. Brain. 2012;135:2809‐2816. 22879644 10.1093/brain/aws190 PMC3437024

