# Supplementary material for: Systemic inflammation and modifiable risk factors for cognitive impairment in older persons: Findings from a British birth cohort
Source: Aging Med (Milton). 2018 Nov 13;1(3):243–8. doi: 10.1002/agm2.12044 (PMC6640037; doi:10.1002/agm2.12044)
Supplement: Supplementary file 1 [file AGM2-1-243-s001.docx]

**Supplementary Table 1: Linear regression analysis between ACE-III aged 69 and serum inflammatory markers at 60-64, adjusted for individual covariates**

| **ACE - III** |  | **CRP at 60-64** | | | | **IL6** | | | | **WCC at 60-64** | | | |
| --- | --- | --- | --- | --- | --- | --- | --- | --- | --- | --- | --- | --- | --- |
|  |  | n | 1st tertile | 2nd tertile | 3rd tertile | n | 1st tertile | 2nd tertile | 3rd tertile | n | 1st tertile | 2nd tertile | 3rd tertile |
| **Unadjusted** | Coefficient | 1416 | Ref | -0.57 | -1.37 | 1426 | Ref | -0.60 | -1.35 | 1432 | Ref | -0.36 | -1.13 |
|  | 95% CI |  |  | -1.30 | -2.11 |  |  | -1.32 | -2.08 |  |  | -1.09 | -1.87 |
|  |  |  |  | 0.16 | -0.62 |  |  | 0.12 | -0.61 |  |  | 0.37 | -0.39 |
|  | p value |  |  |  | <0.01 |  |  |  | <0.01 |  |  |  | <0.01 |
| **Adjusted for sex** | Coefficient | 1431 | Ref | -0.58 | -1.39 | 1425 | Ref | -0.61 | -1.34 | 1432 | Ref | -0.35 | -1.11 |
|  | 95% CI |  |  | -1.31 | -2.14 |  |  | -1.33 | -2.08 |  |  | -1.08 | -1.85 |
|  |  |  |  | 0.15 | -0.64 |  |  | 0.12 | -0.61 |  |  | 0.38 | -0.37 |
|  | p value |  |  |  | <0.01 |  |  |  | <0.01 |  |  |  | 0.01 |
| **Adjusted for BMI** | Coefficient | 1429 | Ref | -0.41 | -1.06 | 1423 | Ref | -0.40 | -1.02 | 1430 | Ref | -0.24 | -0.91 |
|  | 95% CI |  |  | -1.15 | -1.85 |  |  | -1.14 | -1.79 |  |  | -0.97 | -1.66 |
|  |  |  |  | 0.33 | -0.28 |  |  | 0.33 | -0.25 |  |  | 0.50 | -0.16 |
|  | p value |  |  |  | 0.03 |  |  |  | 0.03 |  |  |  | 0.05 |
| **Adjusted for Exercise** | Coefficient | 1396 | Ref | -0.53 | -1.08 | 1390 | Ref | -0.33 | -0.90 | 1397 | Ref | -0.13 | -0.79 |
|  | 95% CI |  |  | -1.25 | -1.83 |  |  | -1.04 | -1.64 |  |  | -0.85 | -1.52 |
|  |  |  |  | 0.20 | -0.34 |  |  | 0.39 | -0.15 |  |  | 0.60 | -0.05 |
|  | p value |  |  |  | 0.02 |  |  |  | 0.06 |  |  |  | 0.09 |
| **Adjusted for Smoking** | Coefficient | 1146 | Ref | -0.53 | -0.96 | 1141 | Ref | -0.60 | -0.84 | 1138 | Ref | -0.24 | -0.29 |
|  | 95% CI |  |  | -1.35 | -1.80 |  |  | -1.41 | -1.68 |  |  | -1.07 | -1.15 |
|  |  |  |  | 0.29 | -0.12 |  |  | 0.22 | -0.01 |  |  | 0.59 | 0.57 |
|  | p value |  |  |  | 0.08 |  |  |  | 0.12 |  |  |  | 0.78 |
| **Adjusted for Alcohol** | Coefficient | 1325 | Ref | -0.51 | -0.95 | 1320 | Ref | -0.64 | -1.03 | 1332 | Ref | -0.26 | -0.68 |
|  | 95% CI |  |  | -1.22 | -1.69 |  |  | -1.35 | -1.76 |  |  | -0.97 | -1.41 |
|  |  |  |  | 0.21 | -0.22 |  |  | 0.07 | -0.30 |  |  | 0.46 | 0.05 |
|  | p value |  |  |  | 0.04 |  |  |  | 0.02 |  |  |  | 0.18 |
| **Adjusted for Education** | Coefficient | 1431 | Ref | -0.15 | -0.44 | 1425 | Ref | -0.54 | -0.69 | n = 1432 | Ref | -0.08 | -0.44 |
|  | 95% CI |  |  | -0.81 | -1.13 |  |  | -1.20 | -1.36 |  |  | -0.75 | -1.12 |
|  |  |  |  | 0.52 | 0.25 |  |  | 0.11 | -0.02 |  |  | 0.58 | 0.23 |
|  | p value |  |  |  | 0.44 |  |  |  | 0.10 |  |  |  | 0.40 |
