# Supplementary material for: Systemic inflammation and modifiable risk factors for cognitive impairment in older persons: Findings from a British birth cohort
Source: Aging Med (Milton). 2018 Nov 13;1(3):243–8. doi: 10.1002/agm2.12044 (PMC6640037; doi:10.1002/agm2.12044)
Supplement: Supplementary file 2 [file AGM2-1-243-s002.docx]

**Supplementary Table 2: Linear regression analysis between visual search speed aged 69 and serum inflammatory markers at 60-64**

| **Visual Search Speed** |  | **Adjusted for search speed at 53 (Complete)** | | | | **Adjusted for prior performance and educational attainment** | | | | **Fully adjusted** | | | | |
| --- | --- | --- | --- | --- | --- | --- | --- | --- | --- | --- | --- | --- | --- | --- |
| **n = 1117** |  | **Coefficient** | **95% CI** | | **p value** | **Coefficient** | **95% CI** | | **p value** | **Coefficient** | **95% CI** | | **p value** |  |
| Visual search speed aged 53 |  | 0.45 | 0.40 | 0.50 | <0.01 |  |  |  |  | 0.44 | 0.39 | 0.49 | <0.01 |  |
| CRP at 60-64 | 1st tertile | Ref |  |  |  | Ref |  |  |  | Ref |  |  |  |  |
|  | 2nd tertile | -5.95 | -14.88 | 2.99 |  | -5.45 | -14.40 | 3.50 |  | -2.92 | -12.18 | 6.33 |  |  |
|  | 3rd tertile | -3.34 | -12.41 | 5.73 | 0.42 | -2.19 | -11.36 | 6.98 | 0.49 | 3.04 | -7.38 | 13.47 | 0.48 |  |
| IL6 at 60-64 | 1st tertile | Ref |  |  |  | Ref |  |  |  | Ref |  |  |  |  |
|  | 2nd tertile | -5.55 | -14.50 | 3.39 |  | -5.35 | -14.30 | 3.60 |  | -4.19 | -13.56 | 5.18 |  |  |
|  | 3rd tertile | -9.92 | -19.01 | -0.83 | 0.10 | -9.35 | -18.48 | -0.23 | 0.13 | -8.25 | -18.95 | 2.46 | 0.32 |  |
| WCC at 60-64 | 1st tertile | Ref |  |  |  | Ref |  |  |  | Ref |  |  |  |  |
|  | 2nd tertile | -5.17 | -14.24 | 3.91 |  | -5.03 | -14.11 | 4.06 |  | -3.40 | -12.74 | 5.93 |  |  |
|  | 3rd tertile | -8.59 | -17.60 | 0.42 | 0.17 | -8.01 | -17.04 | 1.02 | 0.21 | -4.56 | -14.61 | 5.49 | 0.64 |  |
| Educational Attainment by 26 | <O levels | Ref |  |  |  |  |  |  |  | Ref |  |  |  |  |
|  | O levels | 3.10 | -7.29 | 13.50 |  |  |  |  |  | 2.32 | -8.40 | 13.04 |  |  |
|  | >=A levels | 7.73 | -0.70 | 16.16 | 0.19 |  |  |  |  | 7.78 | -1.34 | 16.90 | 0.23 |  |
| Sex |  | 3.46 | -3.97 | 10.89 | 0.36 | 4.47 | -3.08 | 12.01 | 0.25 | 2.43 | -5.41 | 10.27 | 0.54 |  |
| BMI at 60-64 (per SD) |  | 0.45 | 0.40 | 0.50 | <0.01 | -2.25 | -6.28 | 1.79 | 0.27 | -1.23 | -5.63 | 3.16 | 0.58 |  |
| Exercise at 60-64 | Never | Ref |  |  |  | Ref |  |  |  | Ref |  |  |  |  |
|  | 1-4 times | 0.84 | -10.05 | 11.73 |  | -0.25 | -11.27 | 10.78 |  | -0.01 | -11.18 | 11.16 |  |  |
|  | >5 times | 7.21 | -1.72 | 16.14 | 0.28 | 6.14 | -2.90 | 15.18 | 0.38 | 4.54 | -4.74 | 13.81 | 0.61 |  |
| Smoking (Pack Years) at 60-64 | Lifelong Non-smoker | Ref |  |  |  | Ref |  |  |  | Ref |  |  |  |  |
|  | 1-5 pack years | 4.28 | -5.89 | 14.45 |  | 4.29 | -5.88 | 14.46 |  | 5.44 | -4.89 | 15.77 |  |  |
|  | 6-20 pack years | -1.33 | -11.23 | 8.57 |  | -0.97 | -10.89 | 8.94 |  | 1.12 | -9.11 | 11.35 |  |  |
|  | >20 pack years | -5.44 | -15.83 | 4.95 | 0.43 | -3.81 | -14.40 | 6.78 | 0.60 | 0.03 | -11.21 | 11.26 | 0.75 |  |
| Alcohol at 68 | Never | Ref |  |  |  | Ref |  |  |  | Ref |  |  |  |  |
|  | Less than monthly | 3.09 | -13.27 | 19.46 |  | 2.58 | -13.79 | 18.95 |  | 1.65 | -14.80 | 18.09 |  |  |
|  | 2-4 times per month | -4.89 | -17.11 | 7.32 |  | -6.22 | -18.51 | 6.07 |  | -6.93 | -19.32 | 5.46 |  |  |
|  | 2-4 times per week | -1.85 | -12.78 | 9.08 |  | -3.51 | -14.56 | 7.54 |  | -5.10 | -16.45 | 6.26 |  |  |
|  | 4 or more times per week | -2.84 | -13.17 | 7.49 | 0.88 | -5.53 | -16.17 | 5.11 | 0.73 | -6.62 | -17.64 | 4.41 | 0.67 |  |

**Supplementary Table 3: Linear regression analysis between verbal memory aged 69 and serum inflammatory markers at 60-64**

| **Verbal Memory** |  | **Adjusted for verbal memory at 53 (Complete)** | | | | **Adjusted for prior performance and educational attainment** | | | | **Fully adjusted** | | | | |
| --- | --- | --- | --- | --- | --- | --- | --- | --- | --- | --- | --- | --- | --- | --- |
| **n = 1167** |  | **Coefficient** | **95% CI** | | **p value** | **Coefficient** | **95% CI** | | **p value** | **Coefficient** | **95% CI** | | **p value** |  |
| Verbal memory aged 53 |  | 0.67 | 0.62 | 0.71 | <0.01 |  |  |  |  | 0.59 | 0.54 | 0.64 | <0.01 |  |
| CRP at 60-64 | 1st tertile | Ref |  |  |  | Ref |  |  |  | Ref |  |  |  |  |
|  | 2nd tertile | 0.11 | -0.50 | 0.72 |  | 0.18 | -0.43 | 0.79 |  | 0.19 | -0.43 | 0.81 |  |  |
|  | 3rd tertile | -0.36 | -0.99 | 0.26 | 0.31 | -0.18 | -0.80 | 0.44 | 0.53 | -0.09 | -0.79 | 0.61 | 0.67 |  |
| IL6 at 60-64 | 1st tertile | Ref |  |  |  | Ref |  |  |  | Ref |  |  |  |  |
|  | 2nd tertile | -0.15 | -0.76 | 0.46 |  | -0.15 | -0.75 | 0.46 |  | 0.00 | -0.63 | 0.63 |  |  |
|  | 3rd tertile | -0.32 | -0.95 | 0.30 | 0.60 | -0.22 | -0.84 | 0.41 | 0.78 | 0.11 | -0.60 | 0.83 | 0.94 |  |
| WCC at 60-64 | 1st tertile | Ref |  |  |  | Ref |  |  |  | Ref |  |  |  |  |
|  | 2nd tertile | -0.24 | -0.86 | 0.38 |  | -0.21 | -0.82 | 0.41 |  | 0.06 | -0.57 | 0.69 |  |  |
|  | 3rd tertile | -0.24 | -0.85 | 0.38 | 0.69 | -0.13 | -0.75 | 0.48 | 0.80 | 0.41 | -0.26 | 1.09 | 0.43 |  |
| Educational Attainment by 26 | <O levels | Ref |  |  |  |  |  |  |  | Ref |  |  |  |  |
|  | O levels | 1.14 | 0.40 | 1.89 |  |  |  |  |  | 0.89 | 0.14 | 1.64 |  |  |
|  | >=A levels | 1.57 | 0.94 | 2.19 | <0.01 |  |  |  |  | 1.28 | 0.63 | 1.94 | <0.01 |  |
| Sex |  | 0.50 | -0.01 | 1.02 | 0.05 | 0.73 | 0.22 | 1.25 | 0.01 | 0.84 | 0.31 | 1.38 | <0.01 |  |
| BMI at 60-64 (per SD) |  | -0.20 | -0.48 | 0.07 | 0.14 | -0.12 | -0.39 | 0.15 | 0.39 | -0.04 | -0.34 | 0.25 | 0.77 |  |
| Exercise at 60-64 | Never | Ref |  |  |  | Ref |  |  |  | Ref |  |  |  |  |
|  | 1-4 times | 0.71 | -0.04 | 1.46 |  | 0.52 | -0.22 | 1.27 |  | 0.42 | -0.33 | 1.17 |  |  |
|  | >5 times | 1.24 | 0.63 | 1.85 | <0.01 | 1.07 | 0.46 | 1.68 | <0.01 | 1.00 | 0.37 | 1.62 | <0.01 |  |
| Smoking (Pack Years) at 60-64 | Lifelong Non-smoker | Ref |  |  |  | Ref |  |  |  | Ref |  |  |  |  |
|  | 1-5 pack years | -0.21 | -0.90 | 0.48 |  | -0.19 | -0.88 | 0.50 |  | -0.29 | -0.98 | 0.41 |  |  |
|  | 6-20 pack years | -0.56 | -1.23 | 0.12 |  | -0.47 | -1.13 | 0.20 |  | -0.50 | -1.18 | 0.19 |  |  |
|  | >20 pack years | -1.36 | -2.08 | -0.65 | <0.01 | -1.10 | -1.82 | -0.38 | 0.02 | -1.12 | -1.87 | -0.36 | 0.04 |  |
| Alcohol at 68 | Never | Ref |  |  |  | Ref |  |  |  | Ref |  |  |  |  |
|  | Less than monthly | -0.58 | -1.71 | 0.54 |  | -0.67 | -1.79 | 0.44 |  | -0.80 | -1.91 | 0.31 |  |  |
|  | 2-4 times per month | 0.53 | -0.31 | 1.36 |  | 0.37 | -0.46 | 1.20 |  | 0.32 | -0.51 | 1.15 |  |  |
|  | 2-4 times per week | 0.42 | -0.33 | 1.17 |  | 0.21 | -0.54 | 0.96 |  | 0.23 | -0.54 | 0.99 |  |  |
|  | 4 or more times per week | 0.86 | 0.15 | 1.58 | 0.04 | 0.56 | -0.16 | 1.28 | 0.20 | 0.80 | 0.05 | 1.54 | 0.04 |  |
